# Applications of Artificial Intelligence in Health Care Delivery

**DOI:** 10.1007/s10916-023-02018-y

**Published:** 2023-11-17

**Authors:** Joseph Spear, Jesse M. Ehrenfeld, Brian J. Miller

**Affiliations:** 1 University of Oklahoma College of Medicine, Oklahoma City, OK USA; 2 The Medical College of Wisconsin, Milwaukee, WI USA; 3 The Advancing a Healthier Wisconsin Endowment, Milwaukee, WI USA; 4 Division of Hospital Medicine, Department of Medicine, The Johns Hopkins University School of Medicine, 600 N. Wolfe Street Meyer 8-143, Baltimore, MD 21287 USA; 5 American Enterprise Institute, Washington, DC USA

## Abstract

Health care costs now comprise nearly one-fifth of the United States’ gross domestic product, with the last 25 years marked by rising administrative costs, a lack of labor productivity growth, and rising patient and physician dissatisfaction. Policy experts have responded with a series of reforms that have – ironically - increased patient and physician administrative burden with little meaningful effect on cost and quality. Artificial intelligence (AI), a topic of great consternation, can serve as the “wheat thresher” for health care delivery, empowering and freeing both patients and physicians by decreasing administrative burden and improving labor productivity. In this Viewpoint, we discuss three principal areas where AI poses an unprecedented opportunity to reduce cost, improve care, and markedly enhance the patient and physician experience: (1) automation of administrative process, (2) augmentation of clinical practice, and (3) automation of elements of clinical practice.

## Applications of Artificial Intelligence in Health Care Delivery

Health care costs now comprise nearly one-fifth of the United States’ gross domestic products, with the last 25 years marked by rising administrative costs, a lack of labor productivity growth, and rising patient and physician dissatisfaction [1]. Policy experts have responded with a series of reforms that have – ironically - increased patient and physician administrative burden with little meaningful effect on cost and quality. Some programs, such as Accountable Care Organizations have driven consolidation [2] while other long-touted interventions such as comprehensive primary care showed no systematic impact on cost or quality [3]. With decades of reliance on micro policy adjustments not yielding the desired outcomes, now is the time to think differently. Artificial intelligence (AI), a topic of great consternation, can serve as the “wheat thresher” for health care delivery, empowering and freeing both patients and physicians. In this Viewpoint, we discuss three principal areas where AI poses an unprecedented opportunity to reduce cost, improve care, and markedly enhance the patient and physician experience: (1) automation of the mundane, (2) augmentation of human-driven clinical practice, and (3) automation of elements of clinical practice.

### Automation of the Mundane

Nearly two-thirds of physicians suffer from burnout, with many citing electronic health record documentation and inbox messages, prior authorization, and other routine administrative tasks [4]. This collective experience is mirrored in empirical research: a recent time motion study of internal medicine residents demonstrated that only 13% of the average day is spent in face-to-face contact with patients [5] while other work demonstrates that a typical primary care physicians spend 6 h per clinic day writing patient notes [6].

AI offers the opportunity to restore joy in clinical practice through automation of the mundane – or manual, human capital driven tasks. With companies such as Augmedix, DeepScribe and Amazon presenting early attempts at automating clinical notetaking, eventually the burden of recordkeeping should shift from the clinician to technology. Ultimately, clinicians should be able to review and sign AI-generated notes as opposed to spending their limited time during and after patient encounters documenting visits. Early evidence suggests that automation of simple responses to patient messages may not only decrease burden, but also increase satisfaction as AI may be perceived in some circumstances as having greater empathy [7]. Finally, the arduous painful tasks of coding and billing could be similarly automated, saving clinicians from struggling with over 70,000 unique diagnosis codes.

A process that could arguably not be worse for all those involved, prior authorization offers another obvious opportunity for improvement using AI. The average oncology practice has over 6 full-time staff to handle prior authorizations, [8] while physicians generally report filling out an average of 37 prior authorization forms per week with 75% reporting this as a “high” or “extremely high” burden [9]. Using AI to automate the submission of data as the first level of review for some prior authorization processes based upon clear and transparent guidelines could eliminate the need for physician involvement. Eventually, AI could help facilitate near real-time adjudication of prior authorization requests during a clinical visit, freeing up both clinicians and health plan staff to engage in more meaningful, complex reviews.

### Augmentation of human-driven Clinical Practice

AI can transform how clinicians use information and make decisions, driving increasingly personalized care. AI technology [10] drives dynamic displays in Teslas, alerting drivers of potential harm thus facilitating quick decision making and real-time interventions while BlueCruise in Fords facilitates hands-free highway driving. Medical care is no different, requiring split second decisions in an environment of countless informational inputs. AI-driven technology can offer meaningful clinical decision support, enabling clinicians in acute care environments such as intensivists, anesthesiologists, and nurses to manage patients more efficiently and effectively by highlighting concerning trends, gaps in care, or suggesting actions that would otherwise be omitted or delayed.

Technological innovations like magnetic resonance imaging and genetic sequencing have provided clinicians with additional diagnostic tools to customize and personalize care, with AI and automation offering a similar opportunity to upskill clinicians while improving productivity. Through algorithmic pattern recognition, AI applications may more accurately read CT scans, [11] mammography, [12] or pathology slides, [13] catching diagnoses that might have been missed and enhancing the capability of the supervising clinicians. We expect that these tools will, for example, enable pathologists to spend time reviewing suggested diagnoses of routine cases and devote more time to selecting assays, improving overall lab performance, and addressing complex cases. For some cancers, AI tools will eventually be able to suggest treatment options for oncologists to review and then discuss with their patients. This will enhance the consistency of care and accelerate the adoption of newly generated scientific evidence which today often takes decades to come into routine clinical practice.

### Automation of Elements of Clinical Practice

While fully autonomous AI-driven medical care is likely decades away, automation of specific narrow tasks is already underway. Technology like IDx-DR [14] (Digital Diagnostics Inc, Iowa) can streamline screening for diabetic retinopathy without physician interpretation and with such precision that the company holds liability insurance. In resource-limited settings, point-of-care digital cytology with AI is being used for cervical cancer screening [15]. After being trained on 30,493 electroencephalograms, an AI model was able to achieve diagnostic performance similar to human experts [16].

These initial narrow and specific autonomous AI applications offer insight into the future of innovation. For example, as radiological AI applications reach the market, images will be read increasingly by machine learning algorithms with diagnoses being determined with a pre-specified degree of certainty, enabling radiologists to review cases with a low degree of certainty. The transition to automation will be gradual with human practitioners remaining relevant and in the loop supervising the algorithms while finding new avenues to care for patients, requiring physicians to develop the skills to oversee and manage the output and activities of AI-enabled technologies.

### Policy Opportunities

With technological advances comes the fear of the unknown. The wheat thresher was alleged to destroy the agricultural industry and put farmers out of business. In reality, it provided much needed efficiencies in agriculture and allowed farmers to scale and meet increasing demand. AI will play a similar long-term role in that it is an innovative and flexible answer to one of the largest issues facing the healthcare sector: the lack of labor productivity growth coupled with rising administrative costs. Like technological change in order industries, AI may drive some short term costs through implementation, with eventual, significant long-term cost savings.

Important and answerable policy questions remain. Medical licensure for independently practicing products should be avoided. Instead, noting that current U.S. Food and Drug Administration regulatory pathways are ill-suited for complex and rapidly evolving software products, policymakers should consider the creation of a series of voluntary, alternative regulatory pathways fit for software as a medical device, inclusive of AI and software-driven medical devices. Liability remains a question and should be placed with the parties best positioned to mitigate the risk. Policymakers and courts will sort out the gray line between product, clinician, and consumer liability for AI-augmented and AI-driven medical care.

Regardless, the benefits of AI to patients and clinicians must not be overlooked. AI has the potential to transform care delivery augmenting physicians and the healthcare team, leading to the provision of superior care and substantial costs savings, the stated policy goals of many previously failed policy interventions. If the profession panics and fails to capitalize on the positive potential of AI, it will neglect an important opportunity to revolutionize healthcare.

*The views of this paper do not necessarily represent those of the Medicare Payment Advisory Commission or the American Medical Association.*

## Data Availability

No data were used in this study as it is a perspective paper.
